# Obstetric near misses among women with serious mental illness: data linkage cohort study

**DOI:** 10.1192/bjp.2020.250

**Published:** 2021-09

**Authors:** Abigail Easter, Jane Sandall, Louise M. Howard

**Affiliations:** 1 Section of Women's Mental Health, Health Service Research and Population Department, Institute of Psychiatry, Psychology and Neuroscience, King's College London, UK; and Department of Women and Children's Health, Faculty of Life Sciences and Medicine, King's College London, St Thomas’ Hospital, UK; 2 Department of Women and Children's Health, Faculty of Life Sciences and Medicine, King's College London, St Thomas’ Hospital, UK; 3 Section of Women's Mental Health, Health Service Research and Population Department, Institute of Psychiatry, Psychology and Neuroscience, King's College London, UK

**Keywords:** Perinatal psychiatry, comorbidity, integrated care, women's mental health, electronic healthcare records

## Abstract

**Background:**

Investigating obstetric near misses (life-threatening obstetric complications) provides crucial information to prevent maternal mortality and morbidity.

**Aims:**

To investigate the rate and type of obstetric near misses among women with serious mental illness (SMI).

**Method:**

We conducted a historical cohort study, using de-identified electronic mental health records linked with maternity data from Hospital Episode Statistics. The English Maternal Morbidity Outcome Indicator was used to identify obstetric near misses at the time of delivery in two cohorts: (1) exposed cohort – all women with a live or still birth in 2007–2016, and a history of secondary mental healthcare before delivery in south-east London (*n* = 13 570); (2) unexposed cohort – all women with a live or still birth in 2007–2016, resident within south-east London, with no history of mental healthcare before delivery (*n* = 223 274).

**Results:**

The rate of obstetric near misses was 884.3/100 000 (95% CI 733.2–1057.4) maternities in the exposed group compared with 575.1/100 000 (95% CI 544.0–607.4) maternities in the unexposed group (adjusted odds ratio 1.6, 95% CI 1.3–2.0, *P* < 0.001). Highest risks were for acute renal failure (adjusted odds ratio 2.1, 95% CI 1.1–3.8, *P* = 0.022); cardiac arrest, failure or infarction (adjusted odds ratio 2.3, 95% CI 1.1–4.8, *P* = 0.028); and obstetric embolism (adjusted odds ratio 3.1, 95% CI 1.6–5.8, *P* < 0.001).

**Conclusions:**

Findings emphasise the importance of integrated physical and mental healthcare before and during pregnancy for women with SMI.

Mental illness affects around one in four women during early pregnancy.^1^ Women with mental illness, particularly those who experience serious mental illness (SMI; i.e. those in contact with secondary mental health services), are disproportionately affected by a range of adverse foetal and maternal outcomes, including premature birth and low birth weight,^2^ and increased maternal mortality.^3^ As the maternal mortality rate decreases, investigating maternal near misses (or life-threatening obstetric complications) can provide crucial information to guide prevention of maternal deaths and severe morbidity.^4,5^ However, to our knowledge, the risk of obstetric near misses among women with SMI remains unstudied.

## Method

Using linked routine electronic healthcare records on maternity data from hospital admissions and secondary mental healthcare (i.e. specialist in-patient or out-patient mental healthcare for serious or chronic mental illness) in the UK, we aimed to investigate the rate and characteristics of obstetric near misses among women with SMI (i.e. women with a current or history of treatment within secondary mental health services).

### Design and participants

We conducted a historical cohort study, using linked electronic clinical records from Admitted Patient Care Hospital Episode Statistics (HES) and the Clinical Record Interactive Search (CRIS) system. HES is a data warehouse containing records of all patients admitted to National Health Service hospitals in England, and captures data on 97% of all births in England.^6^ CRIS is a unique source of de-identified data on 1.2 million people in contact with mental health services in south-east London.^7^

Maternity records are contained within the HES in-patient data. All babies born in hospital are recorded as a ‘delivery episode’, which refers to a continuous period of hospital care containing a live or still birth. Home births, which account for about 2.1% of births for England and Wales in 2017,^8^ are not included within HES data-sets.

SMI can be defined in different ways, but most definitions include schizophrenia, bipolar disorder and other mental health disorders requiring treatment from secondary mental health services. In this study, given the often severe and enduring nature of these mental health disorders, SMI was defined as women receiving face-to-face treatment from secondary mental healthcare services.

Mild-to-moderate mental health problems are typically treated within primary healthcare services in the UK, e.g. general practices or Improving Access to Psychological Therapies services. Individuals with more severe or complex psychiatric conditions may require referral to secondary mental health services for specialist mental health treatment. These services include support and treatment delivered in the community as well as in hospitals, and include services such as community mental health teams, crisis resolution and home treatment, assertive outreach, and early intervention teams.

This definition therefore includes women with a range of psychiatric diagnoses requiring secondary mental healthcare, rather than restricting the inclusion criteria by diagnoses.

Data on two cohorts of women were extracted: the exposed cohort comprised all women with a delivery episode between January 2007 and March 2016, and a history of secondary mental healthcare either during or before the childbirth episode, in south-east London (women who were referred to mental healthcare for the first-time after childbirth were not included); the unexposed cohort comprised all women with a delivery episode in 2007–2016, resident within the same area, with no history or current use of secondary mental healthcare.

Women were categorised as having a history of receiving secondary mental healthcare if this occurred at any point before and/or during their current pregnancy, recorded in the CRIS system from 2007. Women who were not receiving any mental healthcare or only received mental healthcare outside of secondary mental health services were included within the unexposed cohort.

Some delivery episodes have many delivery records associated with them that are not necessarily identical.^9^ Therefore, the following methods were used to select an individual record to represent the delivery episode. First, all delivery records with identical data (‘full duplicates’) were removed from the data-set (*n* = 1233). Subsequently, if two successive records were separated by more than 30 weeks, then they were considered to relate to different delivery episodes. In total, 1898 episodes were not separated by 30 weeks, and therefore removed as they were considered to relate to the same pregnancy episode. A delivery resulting in more than one baby generates only a single delivery record, and so does not adversely affect the recording of multiple births. This resulted in an overall sample of 13 570 exposed delivery episodes and 223 274 unexposed episodes, after removing data from duplicate and invalid delivery episodes.

### Measures

The English Maternal Morbidity Outcome Indicator (EMMOI) was used to identify obstetric near misses occurring during the delivery episode, by searching International Classification of Disease codes (ICD-10) and Operating Proceedure Codes (OPC-4) among women with and without SMI^10–12^ Adapted from the Australian Maternal Morbidity Outcome Indicator, the EMMOI was developed as a measure of severe maternal morbidity in routine hospital data, and has been previously used to investigate maternal morbidity in HES.^4^

The measure consists of 26 severe morbid childbirth events: 17 diagnoses (i.e. acute abdomen, acute renal failure, acute psychosis, cardiac arrest/failure/infarction, cerebral oedema/coma, disseminated intravascular coagulopathy, cerebrovascular accident, major complications of anaesthesia, obstetric embolism, shock, sickle cell anaemia with crisis, status asthmaticus, status epilepticus, uterine rupture, eclampsia, sepsis and cerebral venous thrombosis) and 9 procedures (i.e. assisted ventilation, curettage with a general anaesthetic, dialysis, evacuation of haematoma, hysterectomy, procedures to reduce blood flow to uterus, re-closure of disrupted Caesarean section wound, repair of bladder/cystostomy and repair of intestine).^4^ Data on maternal deaths occurring during the childbirth episode were extracted from HES.

Mental health data (i.e. psychiatric diagnosis closest to childbirth, psychotropic medication use in the 9 months before childbirth, duration and recent mental health service use) were extracted from mental health records, using structured fields and previously validated language processing applications.^13^

Psychiatric diagnosis closest to childbirth was extracted from mental health records, using a General Architecture for Text Engineering (GATE) software application version 8.6 for Windows (GATE, The University of Sheffield, UK, see www.gate.ac.uk), and was grouped by ICD-10 codes according to the DSM-5: mental disorders due to known physiological conditions (ICD-10 codes F01–F09); mental and behavioural disorders due to psychoactive substance (ICD-10 codes F10–F19); schizophrenia, schizotypal, delusional, and other non-mood psychotic disorders (ICD-10 codes F20–F29); mood [affective] disorders (ICD-10 codes F30–F39); anxiety, dissociative, stress-related, somatoform and other nonpsychotic mental disorders (ICD-10 codes F40–F48); behavioural syndromes associated with physiological disturbances and physical factors (ICD-10 codes F50–F59); disorders of adult personality and behaviour (ICD-10 codes F60–F69); intellectual disabilities (ICD-10 codes F70–F79); pervasive and specific developmental disorders (ICD-10 codes F80–F89); behavioural and emotional disorders with onset usually occurring in childhood and adolescence (ICD-10 codes F90–F98); and unspecified mental disorder (ICD-10 code F99).

Prescribed psychotropic medication was extracted from mental health records by a previously validated natural language processing application, and included the following classes of medication: antipsychotics (included depot antipsychotics), antiepileptics, hypnotics, antidepressants and medications for attention-deficit hyperactive disorder.

Duration of mental health service use was calculated as the number of years since the recorded first contact with secondary mental health services and the delivery episode, and frequency of mental healthcare received was calculated as the number of recorded contacts with mental health services before and during the delivery episode.

Sociodemographic characteristics (i.e. maternal age, ethnicity and Index of Multiple Deprivation (IMD) recorded during the delivery episode) were extracted from HES. IMD is the official measure of relative deprivation for small areas in England, and ranks area from 1 (most deprived area) to 32 844 (least deprived area) on seven domains (income deprivation, employment deprivation, education, skills and training deprivation, health deprivation and disability, crime, barriers to housing and services and living environment deprivation), to produce an overall relative measure of deprivation. Smoking status during pregnancy was not available; therefore, a binary variable of nicotine dependence was created. All women with a clinical diagnosis of nicotine dependence (ICD-10 code F19) recorded in HES during the delivery episode were coded 1, and an absence of nicotine dependence diagnoses was coded 0.

### Statistical analysis

Data were analysed in Stata for Windows version 12.1, after cleaning and validation.^9^ A composite indicator of total obstetric near misses, including all physical health diagnoses and procedures from the EMMOI, was generated. This study aimed to investigate severe obstetric complications of childbirth among women with and without SMI; therefore, acute psychosis recorded during the childbirth episode, which was included within the original EMMOI composite, was not included within the total composite indicator in this analysis of obstetric near misses.

Univariate linear and logistic regressions were conducted to assess differences in sociodemographic characteristics between the exposed and unexposed cohorts. To account for correlations between two or more delivery episodes from a single woman, cluster analysis (using Stata ‘cluster command’, which produces a grouping variable for women based on delivery episodes) was applied.

Clustered univariate and multivariate logistic regression analyses (adjusting for *a priori* determined confounders of ethnicity, maternal age and IMD) were used to examine the rate of individual and composite indicators between groups. A subsequent regression model, with the inclusion of nicotine dependence, was conducted to investigate the mediating role of nicotine dependence.

A subsequent, within-group analysis was conducted to explore potential associations between mental health service use, psychiatric diagnosis, psychotropic medication use in pregnancy and obstetric near misses in the exposed group of women, using clustered logistic and linear regression models. Psyciatric diagnoses were grouped into the following prior to subgroup analysis: psychotic and bipolar disorders (ICD-10 codes F20–F31 and F32.3), depressive disorders (ICD-10 codes F32–F39, excluding F32.3), anxiety disorders (ICD-10 codes F40–F49), and all other psychiatric disorders (ICD-10 codes F0–F19 and F50–F98).

### Missing data

There were 12.5% of missing data for ethnicity and 0.5% of missing data for deprivation level. Complete data were available for all other variables included within the multivariate analysis. Missingness was not associated with any other variables included within the analysis. As the proportion of missing data was small and limited to two variables in the analysis, and the data were considered to be missing at random, a complete-case analysis was conducted.^14^ To avoid identification of women, the results presented are restricted to individual obstetric complications affecting ten or more women; all EMMOI items were included within analyses of composite scores of total obstetric near misses.

### Ethical approval

The authors assert that all procedures contributing to this work comply with the ethical standards of the relevant national and institutional committees on human experimentation and with the Helsinki Declaration of 1975, as revised in 2008. The CRIS system is an approved data-set for secondary analysis, approved by the Oxfordshire Research Ethics Committee (reference 18/SC/0372), this project was approved by the CRIS Oversight Committee (reference 16-049). Section 251 of the NHS Act 2006 approval has been granted for all the above linkages, which allows secondary analysis of de-identified data without informed consent.

## Results

### Sample characteristics

There were 13 570 delivery episodes among 10 216 women with SMI, compared with 223 274 in the unexposed group (*n* = 154 570 women).

Women with SMI were, compared with the unexposed group, younger, more likely to be White, have a diagnosis of nicotine dependence recorded during the delivery episode and live in more deprived neighbourhoods (Table 1). Among the exposed cohort, mood disorders (ICD-10 codes F30–F39; *n* = 3294; 39.2%) and anxiety disorders (ICD-10 codes F40–F48; *n* = 1954; 23.2%) were the most frequently recorded primary psychiatric diagnoses, followed by mental disorder due to substance use (ICD-10 codes F10–F19; 12.5%; *n* = 1050); behavioural syndromes associated with physiological disturbances and physical factors, including eating disorders and non-organic sleep disorders (ICD-10 codes F50–F59; 8%; *n* = 676); disorders of adult personality and behaviour (ICD-10 codes F60–F69; 5.2%; *n* = 446); psychotic disorders (ICD-10 codes F20–F29; 5.1%; *n* = 433); behavioural and emotional disorders, with onset usually occurring in childhood (ICD-10 codes F90–F98; 5.1%; *n* = 445); and other diagnoses, including intellectual disabilities (ICD-10 codes F01–F09 and F70–F89; 1.4%; *n* = 119).

**Table 1:** Sample characteristics

	*n*	Exposed cohort	*n*	Unexposed cohort
Age, years, mean (s.d.)	13 570	28.9 (6.4)	223 274	31.1 (5.5)
Ethnicity, *n* (%)				
White		6759 (49.8)		100 508 (45.0)
Mixed or multiple		489 (3.6)		7335 (3.3)
Asian/Asian British		639 (4.7)		18 908 (8.5)
Black/African/Caribbean/Black British	13 570	2851 (21.0)	223 274	51 149 (22.9)
Other ethnic group		829 (6.1)		16 542 (7.4)
Not stated		2003 (14.8)		28 812 (12.9)
Nicotine dependence in pregnancy, *n* (%)				
No^a^		12 266 (90.4)		219 072 (98.1)
Yes	13 570	1303 (9.6)	223 274	4202 (1.9)
Index of Multiple Deprivation, *n* (%)				
Most deprived 10%		1117 (8.3)		15 461 (7.0)
Most deprived 10–30%		6986 (51.8)		97 554 (43.9)
Most deprived 30–50%	13 490	3028 (22.4)	222 179	54 986 (24.7)
Least deprived 30–50%		1258 (9.3)		25 505 (11.5)
Least deprived 10–30%		827 (6.1)		20 191 (9.1)
Least deprived 10%		274 (2.0)		8482 (3.8)

a. An absence of a nicotine dependence diagnosis recorded during the delivery episode was coded as ‘no’.

On average, women's first contact with secondary mental healthcare was 3.9 years (s.d. 2.8) before the delivery episode, with a mean of 26.6 (s.d. 68.9) face-to-face contacts with secondary mental health services. In the year before the delivery episode, 42.3% (*n* = 5734) received secondary mental healthcare, 2.8% (*n* = 384) of whom had an in-patient admission; 23.4% (*n* = 3181) were prescribed psychotropic medications during pregnancy.

### Obstetric near misses

In total, 1404 (0.59%) of women had one or more obstetric near miss during childbirth: 1284 (0.58%) women in the unexposed group compared with 120 (0.88%) women in the exposed group. For the majority of these women (*n* = 1304, 92.9%) one EMMOI near miss indicator was recorded in their delivery notes, with a small minority of women experiencing two or more obstetric near misses during the same childbirth (*n* = 100, 7.1%). Women with SMI had a greater risk of an obstetric near miss during childbirth (884.3 per 100 000 maternities, 95% CI 733.2–1057.4) than the unexposed group (578.1 per 100 000 maternities, 95% CI 544.0–607.4) (odds ratio 1.5, 95% CI 1.3–1.9, *P* < 0.001). The exposed group were at greater risk of sepsis (odds ratio 1.5, 95% CI 1.0–2.4, *P* = 0.040); acute renal failure (odds ratio 1.9, 95% CI 1.1–3.5, *P* = 0.033); cardiac arrest, failure or infarction (odds ratio 2.2, 95% CI 1.1–4.4, *P* = 0.021); and obstetric embolisms (odds ratio 2.5, 95% CI 1.4–4.6, *P* = 0.003) during childbirth, compared with the unexposed group (Table 2). There were no maternal deaths recorded in HES during the childbirth episode in either group.

**Table 2:** Clustered logistic regression of obstetric near misses among women with and without serious mental illness^a^

	Unadjusted odds ratio (95% CI), *P-*value	Adjusted odds ratio (95% CI), *P-*value^b^
Morbid events diagnosis		
Acute renal failure	1.9 (1.1–3.5), *P* = 0.033	2.1 (1.1–3.9), *P* = 0.022
Cardiac arrest, failure or infarction	2.2 (1.1–4.4), *P* = 0.021	2.3 (1.1–4.8), *P* = 0.028
Obstetric embolism	2.5 (1.4–4.6), *P* = 0.003	3.1 (1.6–5.8), *P* = 0.001
Sickle cell anaemia with crisis	2.3 (1.1–4.6), *P* = 0.022	2.1 (0.9–4.3), *P* = 0.051
Uterine rupture	0.9 (0.4–2.0), *P* = 0.882	1.2 (0.5–2.5), *P* = 0.708
Eclampsia	1.1 (0.5–1.9), *P* = 0.960	0.9 (0.5–1.9), *P* = 0.969
Sepsis	1.5 (1.0–2.4), *P* = 0.040	1.6 (0.9–2.4), *P* = 0.051
Morbid events procedures		
Assisted ventilation	1.9 (0.9–4.1), *P* = 0.112	1.9 (0.8–4.5), *P* = 0.126
Evacuation of haematoma	1.2 (0.6–2.3), *P* = 0.639	1.4 (0.7–2.8), *P* = 0.346
Re-closure of disrupted Caesarean section	1.6 (0.8–3.6), *P* = 0.209	2.0 (0.9–4.6), *P* = 0.080
Repair of bladder or cystostomy	1.5 (0.7–3.6), *P* = 0.310	1.6 (0.6–3.9), *P* = 0.325
Morbid events composite		
Total obstetric near misses	1.6 (1.3–2.0), *P* < 0.001	1.7 (1.4–2.1), *P* < 0.001
Total obstetric near miss procedures	1.4 (0.9–2.0), *P* = 0.078	1.6 (1.1–2.4), *P* = 0.015
Total obstetric near miss events	1.5 (1.3–1.9), *P* < 0.001	1.7 (1.4–2.2), *P* < 0.001

a. Regression results for individual morbid diagnoses and procedures are only shown where ten or more women were identified. Composite scores include all English Maternal Morbidity Outcome Indicator diagnoses and procedures.

b. Adjusted for ethnicity, maternal age and deprivation.

**Fig. 1:**
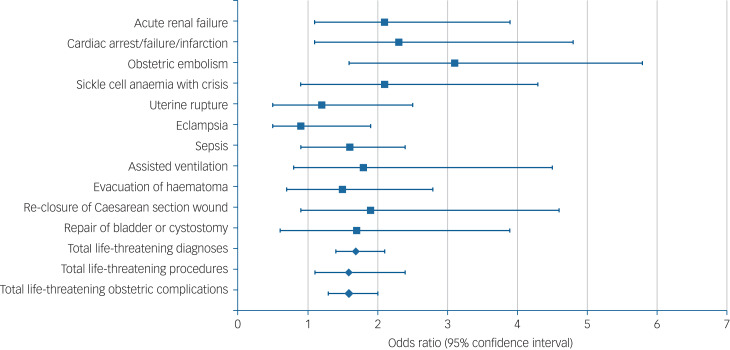
Comparison of obstetric near misses among women with and without serious mental illness. Regression results for individual morbid diagnoses and procedures are only shown where ten or more women were identified. Composite scores include all English Maternal Morbidity Outcome Indicator diagnoses and procedures. Adjusted for ethnicity, maternal age and deprivation.

After adjusting for maternal sociodemographic characteristics, the overall risk of an obstetric near miss remained higher for women with SMI (adjusted odds ratio 1.6, 95% CI 1.3–2.0, *P* < 0.001) (Fig. 1). The inclusion of nicotine dependence as a potential mediator in the multivariate model did not affect the associations between maternal mental illness and total obstetric near misses (adjusted odds ratio 1.7, 95% CI 1.4–2.2, *P* < 0.001), but slightly attenuated associations between exposure group and cardiac arrest (adjusted odds ratio 2.1, 95% CI 0.9–4.7, *P* = 0.057) (Table 2).

Duration of mental healthcare before the delivery episode was higher among women who had an obstetric near miss (mean 4.7 years, s.d. 2.9) compared with women who did not (mean 3.9 years, s.d. 2.8): B_1_ = 0.7, 95% CI 0.2–1.3, *P* = 0.008. Among women with SMI, obstetric near misses were not associated with psychotropic medication use during pregnancy (odds ratio 1.1, 95% CI 0.8–1.7, *P* = 0.534), recent contact with psychiatric services in the year before pregnancy (odds ratio 0.7, 95% CI 0.5–1.1, *P* = 0.106), or alcohol or substance misuse in the year before the delivery episode (odds ratio 0.5, 95% CI 0.2–1.3, *P* = 0.130) (Table 3). Although not statistically significant, the odds ratio for a near misses were found to be higher among women diagnosed with psychotic and bipolar disorders (odds ratio 1.6, 95% CI 0.7–3.8, *P* = 0.246) and those with mood disorders (odds ratio 1.6, 95% CI 0.9–2.8, *P* = 0.132), compared with women diagnosed with anxiety disorders (odds ratio 1.2, 95% CI 0.6–2.5, *P* = 0.542).

**Table 3:** Clinical characteristics among women with serious mental illness, with and without an obstetric near miss

	Obstetric near miss (*n* = 120)	No near miss (*n* = 13 450)	Odds ratio/coefficient^a^ (95% CI), *P-*value
Mental healthcare in the year before delivery episode, *n* (%)			
No	78 (65.0)	7758 (57.7)	0.7 (0.5–1.1), *P* = 0.106
Yes	42 (35.0)	5692 (42.3)	
Psychotropic medication prescribed in pregnancy, *n* (%)			
No	89 (74.2)	10 299 (76.6)	1.1 (0.8–1.7), *P* = 0.534
Yes	31 (25.8)	3150 (23.4)	
Duration since first contact with mental health services before childbirth, years, mean (s.d.)	4.7 (2.9)	3.9 (2.8)	0.7 (0.2–1.3), *P* = 0.008
Frequency of psychiatric care received before childbirth, mean number of contacts (s.d.)	21.5 (56.4)	26.6 (69.0)	−5.2 (−15.3 to −4.9), *P* = 0.316
Psychiatric diagnosis, *n* (%)			
Psychotic and bipolar disorders	8 (6.7)	721 (5.4)	1.6 (0.7–3.8). *P* = 0.246
Depressive disorders	31 (25.8)	2914 (21.7)	1.6 (0.9–2.8), *P* = 0.132
Anxiety disorders	16 (13.3)	1904 (14.2)	1.2 (0.6–2.5), *P* = 0.542
All other psychiatric disorders	18 (15.0)	2662 (19.8)	Reference group
No formal psychiatric disorder/missing	47 (39.2)	5249 (39.0)	
Alcohol or substance misuse in the year before delivery episode, *n* (%)			
No	Value suppressed	12 517	0.5 (0.2–1.3), *P* = 0.130
Yes	<10	933	

a. Odds ratios are displayed for categorical outcomes (i.e. mental healthcare before delivery episode, psychotropic medication and psychiatric diagnosis) and unstandardised beta coefficients are displayed for continuous outcomes (i.e. duration since first contact and frequency of psychiatric care).

## Discussion

This study provides evidence that SMI is associated with an increased risk of obstetric near misses during childbirth, which persisted after accounting for age, ethnicity and socioeconomic status. Although relatively rare, affecting less than 1% of the whole cohort of women in this study, serious complications of childbirth substantially increase the risk of maternal mortality, and have potential long-term implications for maternal and child health. The odds of an obstetric near miss were 1.6 (95% CI 1.3–2.0) higher among women with SMI. This was particularly the case for women with a longer duration of mental health service contact, suggesting that more severe or enduring mental illness may contribute to an overall increase in risk of obstetric near misses. No maternal deaths were recorded during childbirth in this cohort, a longer-term follow-up period would allow for further investigation of the long-term impact and outcomes for women.

We found no evidence that psychotropic medication use during pregnancy or recent contact with mental healthcare services increased the rate of obstetric near misses among women with SMI. The rate of obstetric near misses among women diagnosed with bipolar and psychotic disorders, and mood disorders was higher compared with women diagnosed with anxiety disorders. This finding was not statistically significant, given the small sample size in subgroup analysis, but it suggests that there may be differential effects of psychiatric diagnoses. Further research with larger sample sizes will be helpful in determining the impact of different diagnostic categories on the risk of obstetric near misses among women with mental illness.

Excess morbidity and mortality among individuals with mental illness is well documented, and life expectancy is approximately 10–20 years less that the general population, among both men and women.^15^ Many risk factors contribute to this staggering health disparity. A recent evidence synthesis identified five key modifiable risk factors for physical diseases in those with mental illness (i.e. smoking, excessive alcohol consumption, sleep disturbance, physical inactivity and dietary risks), which should be targeted in multidisciplinary interventions.^16^

Smoking during pregnancy is a common source of pregnancy-related morbidity for women and their infants.^17^ Smoking prevalence is three times higher among those with mental illness,^18^ and continuation during pregnancy has been shown to be considerably more common among women with mental illness.^19^ In this study, nicotine dependence was found to mediate the risk of cardiac arrest during pregnancy, and was five times higher among women with SMI. Around 10% of pregnant women in the UK smoke at the time of delivery,^20^ and therefore the rate of recorded nicotine dependence is likely to be lower than the smoking prevalence in this sample, and may underestimate the effect of smoking on obstetric complications. As a leading cause of maternal and neonatal morbidity and mortality, access to effective smoking cessation interventions for pregnant women with mental disorders is vital. A recent randomised controlled trial found that smoking cessation interventions can be effective for people with SMI, if tailored to this population.^21^

It is well established that obesity has also been shown to increase the risk of several potentially adverse maternal outcomes, including cardiac problems, obstetric embolism and infection,^22^ conditions found here to be more likely in pregnant women with SMI. Empirical studies have highlighted an increased prevalence of obesity among women with mental disorders, including in pregnancy.^23^ Data on pregnancy weight was unfortunately not available for this historical cohort study. However, given the potential importance of risk factors such as smoking and obesity, these findings further highlight the importance of better recording of this data in electronic healthcare records and cross-data linkages in the UK, to better understand their contribution in future research.

In addition to the contribution of such health disparities, maternal mortality inquiries have highlighted ‘diagnostic overshadowing’ as a potential factor in maternal deaths, where women who died of physical complications had their symptoms misattributed to mental illness.^24,25^ It is possible that this also played a role here. Complications that were particularly prevalent among women with mental illness (e.g. obstetric embolism and sepsis) are characterised by vague symptoms, sudden onset or altered mental state, which may increase the risk of misattribution, delayed diagnosis or treatment. Indeed, it has been reported that suboptimal intrapartum care and inadequate prenatal care may contribute to the risk of severe obstetric complications among women with mental illness who have not received mental healthcare.^26^

The reduction of maternal morbidity and mortality is a key international development goal. Effective interventions targeting vulnerable groups, such women with SMI, are vital to achieving this goal. A multimorbidity life-course approach to prevention and treatment of illness, which integrates both physical and mental health,^16^ is also crucial to addressing the issues outlined above at critical life stages, such as preconception and pregnancy. The importance of supporting good preconception maternal health is being increasingly recognised to improving maternal physical and mental health before pregnancy.^27^ In addition, given the disparities in access to family planning among women with SMI, routinely incorporated preconception care within routine appointments for all women with SMI may help reduce adverse obstetric outcomes in this group.

Few studies have investigated obstetric near misses among women with SMI, using linked mental health and childbirth records, adjusted for sociodemographic characteristics and nicotine dependence in pregnancy. Information on maternal mental illness is limited in confidential enquiries into maternal death, because of lack of access to psychiatric healthcare records.^24^ Therefore, this unique data linkage, including detailed data from physical and mental health services, has enabled us to investigate women who are typically under investigated in clinical studies. Limitations include the quality of data recorded in HES, although data validation checks were conducted before analysis, to minimise bias.^10^ Although the overall sample size in this study was large, given the rarity of the outcomes under investigation, this may have led to the study being underpowered to detect differences in subgroup analyses. This may have led to the analysis missing a very large signal in one group and overcalling a non-signal in another. The EMMOI is a useful measure of severe maternal morbidity in healthcare records; however. the measure does not include postpartum haemorrhage, a leading cause of maternal death in UK, as diagnostic data could not be validated in HES. In addition, data were not available on some potentially important confounding and mediating variables; for example, maternal body mass index data and pre-existing health conditions and complications were not available in this linked data-set. Finally, this study was limited to women in south-east London, so the findings may not be generalisable to other populations. Further studies are needed to examine the wide range of factors that may mediate the relationship between maternal mental illness and obstetric near misses.

In conclusion, we report a link between SMI and an increased rate of obstetric near misses during childbirth. More research is needed to fully understand the role of known modifiable risk factors and healthcare provision to the increased risk of obstetric near misses among women with SMI. Nevertheless, these findings do highlight the importance of integrated physical, maternal and mental healthcare before and during pregnancy for women with SMI. Providing sensitive preconception care, which targets known modifiable risk factors, and is integrated into routine healthcare appointments for all women with SMI who are of childbearing age, may help improve obstetric outcomes in this group. Future research should seek to determine the most effective models of care to reduce the persistent health inequalities observed among pregnant women with SMI.

## Data Availability

The data that support the findings of this study are available from the National Institute for Health Research (NIHR) Maudsley Biomedical Research Centre. The Clinical Record Interactive Search system has been developed for use within the NIHR Maudsley Biomedical Research Centre. It provides authorised researchers with regulated, secure access to anonymised information extracted from the South London and Maudsley NHS Foundation Trust electronic clinical records system.
